# Amyloid Beta Resistance in Nerve Cell Lines Is Mediated by the Warburg Effect

**DOI:** 10.1371/journal.pone.0019191

**Published:** 2011-04-26

**Authors:** Jordan T. Newington, Andrea Pitts, Andrew Chien, Robert Arseneault, David Schubert, Robert C. Cumming

**Affiliations:** 1 Department of Biology, The University of Western Ontario, London, Ontario, Canada; 2 Cellular Neurobiology Laboratory, Salk Institute, La Jolla, California, United States of America; Tokyo Medical and Dental University, Japan

## Abstract

Amyloid beta (Aβ) peptide accumulation in the brains of patients with Alzheimer's disease (AD) is closely associated with increased nerve cell death. However, many cells survive and it is important to understand the mechanisms involved in this survival response. Recent studies have shown that an anti-apoptotic mechanism in cancer cells is mediated by aerobic glycolysis, also known as the Warburg effect. One of the major regulators of aerobic glycolysis is pyruvate dehydrogenase kinase (PDK), an enzyme which represses mitochondrial respiration and forces the cell to rely heavily on glycolysis, even in the presence of oxygen. Recent neuroimaging studies have shown that the spatial distribution of aerobic glycolysis in the brains of AD patients strongly correlates with Aβ deposition. Interestingly, clonal nerve cell lines selected for resistance to Aβ exhibit increased glycolysis as a result of activation of the transcription factor hypoxia inducible factor 1. Here we show that Aβ resistant nerve cell lines upregulate Warburg effect enzymes in a manner reminiscent of cancer cells. In particular, Aβ resistant nerve cell lines showed elevated PDK1 expression in addition to an increase in lactate dehydrogenase A (LDHA) activity and lactate production when compared to control cells. In addition, mitochondrial derived reactive oxygen species (ROS) were markedly diminished in resistant but not sensitive cells. Chemically or genetically inhibiting LDHA or PDK1 re-sensitized resistant cells to Aβ toxicity. These findings suggest that the Warburg effect may contribute to apoptotic-resistance mechanisms in the surviving neurons of the AD brain. Loss of the adaptive advantage afforded by aerobic glycolysis may exacerbate the pathophysiological processes associated with AD.

## Introduction

Alzheimer's disease (AD) is a complex neurodegenerative condition, and is the most common form of dementia among the elderly. Currently, there is no cure for the disease and treatment options remain limited. AD is characterized at the histopathological level by widespread nerve cell death, synaptic loss and the accumulation of intracellular neurofibrillary tangles (NFT) and extracellular plaques within the brain [1]. These plaques are primarily composed of amyloid β-peptide (Aβ), a 40–42 amino acid peptide derived from the proteolytic cleavage of the amyloid precursor protein (APP) [2], [3], [4]. A prevalent theory in the field is that AD is caused primarily by Aβ deposition within the brain, which leads to an increased production of reactive oxygen species (ROS), oxidative damage, mitochondrial dysfunction and cell death [5], [6], [7], [8], [9]. Interestingly, some populations of cells within the brain survive by becoming resistant to Aβ toxicity. Immunohistochemical analysis of brain tissue from individuals that died without any history of dementia has revealed that up to 40% of the autopsied samples had significant plaque accumulation [10], [11]. While difficult to study *in vivo*, it is possible to examine amyloid resistance in cultured nerve cells. Clonal nerve cell lines selected for resistance to Aβ toxicity exhibit increased resistance to a wide array of neurotoxins including glutamate, hydrogen peroxide and rotenone, suggesting that these cells have acquired a common resistance mechanism to survive exposure to environmental stresses [7], [12], [13]. Initial studies revealed that Aβ-resistant cells upregulate several antioxidant and glycolytic enzymes [12], [14], [15]. However, further investigation into these survival mechanisms is necessary for a greater understanding of Aβ-sensitivity and resistance.

### Aerobic glycolysis (the Warburg effect) in cancer

In normal nerve cells glucose is converted to pyruvate through a number of steps within the cytosol. In the presence of oxygen, pyruvate is converted into acetyl-Coenzyme A (acetyl-CoA), by pyruvate dehydrogenase (PDH) within the mitochondria. Acetyl-CoA is subsequently fed into the tricarboxcylic acid cycle (TCA cycle), ultimately producing energy via oxidative phosphorylation. In an environment lacking oxygen cells must depend on glycolysis whereby pyruvate is converted into lactate by lactate dehydrogenase subunit A (LDHA), resulting in increased lactate production. Hypoxia inducible factor 1 (HIF-1), a transcription factor induced in hypoxic microenvironments, mediates the critical cellular metabolic adaptation to hypoxia through activation of several glycolytic genes including *ldha* [16], [17]. In addition to mediating the increased conversion of pyruvate to lactate, HIF-1 has recently been shown to actively suppress mitochondrial respiration by directly upregulating the expression of the gene encoding pyruvate dehydrogenase kinase 1 (PDK1) [18], [19]. PDK1 phosphorylates and inhibits PDH, thereby acting as a molecular switch between glycolysis and aerobic respiration to meet cellular ATP needs. Initially HIF-1 was believed to be a transcription factor involved in mediating the cellular metabolic adaptation to hypoxia, however it has more recently been shown to be active in normoxic conditions, such as vascularised cancer tissues, suggesting an addition role for the transcription factor [20], [21].

 Enhanced glycolysis and increased lactate production is a common property of invasive cancers and its upregulation in cancer may result in the suppression of apoptosis [22], [23]. The initial upregulation of glycolysis in tumors is believed to be triggered by a hypoxic microenvironment and HIF-1 activity. However, despite increasing oxygen availability the glycolytic phenotype persists [21], [24]. This phenomenon has been termed the Warburg effect or aerobic glycolysis [22], [25]. In addition to upregulation of glycolysis, cancer cells decrease the flux of pyruvate through the mitochondria via upregulation of PDK, and the inhibition of PDH [22], [26], [27]. This shift in metabolism causes a drop in both mitochondrial oxygen consumption and associated ROS production [22]. Therefore, lower levels of mitochondrial activity lead to a decrease in both ROS production and the propensity of mitochondria to depolarize; two events that trigger apoptosis. The Warburg effect is believed to provide a selective advantage for the survival and proliferation of tumorigenic cells however it has rarely been examined in other cellular contexts [22], [23].

### Aerobic glycolysis in AD

Recent studies using PET imaging revealed a strong spatial correlation between aerobic glycolysis and Aβ deposition in the brains of AD patients [28]. Additionally, activities of the glycolytic enzymes pyruvate kinase (PK), and lactate dehydrogenase A (LDHA), are elevated in the frontal and temporal cortex of patients with AD [29]. In contrast, the reductions of various mitochondrial enzymes involved in cellular respiration have been reported in the AD brain [30], [31], [32]. Interestingly, Aβ-resistant nerve cells *in vitro* exhibit increased glucose uptake and flux through the glycolytic pathway [14]. Aβ-resistant cells are also highly sensitive to glucose deprivation suggesting that the altered glycolytic metabolism in these cells may mediate Aβ resistance. However, it is unknown if Aβ resistant cells repress mitochondrial respiration and rely primarily on glycolysis for their energy needs.

Evidence to suggest a Warburg effect may exist in AD is supported by the observation that elevated levels of HIF-1α are detected in cultured Aβ resistant cells and in the brains of AD transgenic mice compared to controls [14]. Additionally, stabilization of HIF-1α and an increase in HIF-1 activity protects cortical neurons from Aβ toxicity [14]. Potentially, HIF-1 may act in a manner similar to cancer cells by upregulating LDHA and PDK in Aβ resistant cells and surviving neurons of the AD brain. Elevated mitochondrial-derived ROS is strongly linked to Aβ induced death [6], [7], [12]. The impairment of mitochondrial metabolism in AD has been well documented and it is possible that a decrease in the flux of pyruvate through the mitochondria in Aβ resistant cells results in decreased mitochondrial respiration and ROS production similar to cancer cells [22], [33], [34]. An increased reliance on glycolysis for production of ATP may give surviving cells an advantage in the hostile environment of the AD brain. Additionally, decreased mitochondrial activity may attenuate the release of apoptogenic factors [22], [35]. Taken together, the increase in glycolysis and lactate production and/or repression of mitochondrial activity may play a role in the protection of neurons against Aβ toxicity in AD, although this hypothesis has never formally been examined.

To investigate if a Warburg effect exists in Aβ resistant cells, and if this contributes to their resistance against Aβ toxicity, two nerve like cell lines, PC12 and B12, and their Aβ resistant derivatives were selected and characterized. Here we show that nerve cell lines which are resistant to Aβ, break down glucose in a manner reminiscent of cancer cells. Western blot analysis revealed increased levels of PDK1 in Aβ resistant cells exposed to Aβ compared to parental sensitive cells. Aβ resistant cells showed higher levels of LDHA activity and also generated higher levels of lactic acid compared to sensitive cells. Chemical and genetic inactivation of either LDHA or PDK1 promoted increased cell death in Aβ-resistant cells following Aβ exposure. These findings indicate that a shift in metabolism to rely heavily on glycolysis for energy needs, may provide nerve cells with a mechanism to survive Aβ accumulation within the AD brain.

## Methods

### Materials

Cell culture reagents including Dulbecco's modified Eagles medium (DMEM), penicillin/streptomycin P/S, DMEM without phenol red and Dulbecco's phosphate buffered saline (DPBS, 1X) were purchased from Biowhittaker (Walkersville, MD, USA). Dialyzed fetal bovine serum (FBS) and horse serum (HS) were obtained from PAA Laboratories Inc. (Etobicoke, ON, Canada). OPTIMEM I (1X) and TrypLE Express (1x) were obtained from Invitrogen (Carlsbad, CA, USA). Amyloid beta (Aβ) peptide (25–35) was purchased from California peptide research (San Francisco, CA, USA). Poly-D-lysine, L-Lactic dehydrogenase solution Type II, L(+)-lactic acid∼98%, β-Nicotinamide adenine dinucleotide (NAD^+^), β-Nicotinamide adenine dinucleotide reduced disodium salt (NADH), sodium pyruvate, potassium hydroxide, perchloric acid, sodium oxamate, dichloroacetic acid (DCA) ≥99%, Bisbenzimide (Hoechst), Poly-D-lysine, puromycin, dimethyl formamide, 3-(4,5-dimethlythiazol-2-yl)-2,5-diphenyl tetrazolium bromide (MTT) were all purchased from Sigma (St. Louis, MO, USA). G418 sulfate was purchased from Calbiochem (EMD Chemicals Inc., Darmstadt, Germany). Mitotracker Red CM-H2XRos (Molecular Probes) was purchased from Invitrogen (Carlsbad, CA, USA).

### Cell culture

The PC12 and B12 immortalized nerve cell lines and their Aβ resistant derivatives were obtained from Dr. David Schubert (The Salk Institute, La Jolla, CA) and cultured as previously described [7], [12]. The PC12 cell lines used in this study are a subclone of a cell line originally derived from a rat pheochromocytoma [36] and were grown in DMEM supplemented with 10% fetal bovine serum, 5% horse serum, and 1% penicillin/streptomycin. The B12 central nervous system cells are an immortalized clonal cell line derived from a nitrosoethylurea induced brain tumor in rats [37] and were grown in DMEM supplemented with 10% FBS, and 1% P/S. The PC12 and B12 Aβ resistant cell lines were isolated following 4 months growth in the presence of Aβ and subsequent cloning [7], [12]. Prior to experimentation the Aβ resistant clones were re-selected in 20 µM Aβ (25–35) for two weeks. The Aβ peptide (25–35) was dissolved in water at 1 mg/ml, left overnight at room temperature to promote fibril formation and subsequently stored at −20°C.

### LDHA assay

The activity of LDHA was determined spectrophotometrically. PC12 and B12 cells were plated at an appropriate density to achieve 70–80% confluency before exposure to Aβ_25–35_ (20 µM). Aβ-treated and untreated PC12 and B12 cells were washed twice in DPBS and harvested in lysis buffer (50 mM Tris pH 7.5, 2% SDS and 1 mM PMSF) at 24 hr and 48 hr. Following a freeze/thaw cycle, protein extracts were quantified by a Lowry Assay (Bio-Rad, Richmond, CA, USA). LDHA catalyzes the reversible reduction of pyruvate to lactate, using NADH as a co-substrate. The activity of LDHA in each sample was determined by measuring the change in absorbance as a result of the oxidation of NADH at a wavelength of 340 nm (37°C) as previously described [38] and standardized to overall protein concentration.

### Lactate assay

Lactate levels in the extracellular fluid of Aβ treated and untreated PC12 and B12 parental and resistant lines were measured spectrophotometrically [39]. PC12 and B12 cells were plated on 35 mm dishes at an appropriate density to achieve 70–80% confluency following an overnight incubation. One day after seeding the media was aspirated, the cells were washed with DPBS and the media was replaced with OPTIMEM I media. Aβ_25–35_ 20 µM) was also added to the test dishes immediately after changing the media to OPTIMEM I. Following 24 hr and 48 hr incubation lactate released into the medium was measured. One tenth volume of 70% perchloric acid (PCA) was added to the harvested media, and samples were put on ice for 10 minutes. Precipitated proteins were removed by centrifugation, and the acidified supernatant was neutralized by adding the appropriate amount of 10 M KOH to achieve pH 7.5. Lactate levels in the samples were measured spectrophotometrically as described previously [39] and absolute amounts were determined by comparison to a lactate standard curve.

### Immunoblot Analysis

To assess absolute level of LDHA and PDK1, treated (20 µM Aβ_25–35_) and untreated PC12 and B12 cells from subconfluent cultures were washed twice in cold DPBS and harvested in a Tris extraction buffer (50 mM Tris pH 7.5, 2% SDS and 1 mM PMSF) at 24 hr and 48 hr. Protein extracts were quantified by a Lowry assay, resolved by 12% SDS PAGE and electroblotted onto PVDF membrane (Bio-Rad Richmond, CA, USA) [15]. Membranes were probed with the following antibodies: polyclonal anti-LDHA (1∶1000; Cell Signaling, Danvers, MA, USA), polyclonal anti-PDK1 (1∶1000; Stressgen, San Diego, CA, USA) and a monoclonal anti-β-actin (1∶2000; Cell Signaling, Danvers, MA, USA) followed by incubation with an appropriate horseradish peroxidase (HRP) -conjugated secondary antibody (Bio-Rad, Richmond, CA, USA). The blots were developed using Pierce ECL western blotting substrate (Thermo Scientific, Rockford, IL, USA) and visualized with a Bio-Rad Molecular Imager (ChemiDoc XRS, Bio-Rad, Richmond, CA, USA). Densitometric analysis was performed using Image J software. Band densities were standardized against β-actin, and the ratio of LDHA/PDK1-specific bands relative to the β-actin band was determined. Relative intensity was calculated by comparing the LDHA/or PDK1/β-actin ratio of the resistant lines to the same ratio in the parental cell line.

### Cytotoxicity assay

Aβ induced cell cytotoxicity (cell viability) was assessed by a modified MTT assay [7], [12], [40]. The MTT assay measures the reduction of the tetrazolium salt MTT to a colored formazan in living cells [7], [12]. Cells were seeded (3 × 10^3^cells/well) in a 96 well microtiter plate and, following overnight incubation, Aβ_25–35_ was added to the test wells at a concentration of 20 µM. Neutralized DCA (2.5 mM), an inhibitor of PDK1, or oxamate (20 mM), an inhibitor of LDHA, were also added to the appropriate test wells. Following 48 hr incubation 10 µl of MTT stock (2.5 mg/ml dissolved in DPBS) was added to each well, and plates were incubated again, for 4 hours, then 100 µl of solubilization solution (20% SDS in 50% dimethyl formamide pH 4.8) was added to each well and plates were rocked at room temperature overnight. The following day plates were read on a microplate reader (BioRad Model 3550) using 595 nm as the test wavelength and 655 nm as the reference wavelength. The percent viability was calculated from the mean absorbance of the treated cells divided by the mean absorbance of the control cells and multiplied by 100%. Aβ induced cytotoxicity was also assessed by a trypan blue exclusion assay. Cells were seeded in 12-well dishes and following overnight incubation Aβ_25–35_ was added to the test wells at a concentration of 20 µM. Neutralized DCA (2.5 mM) or oxamate (20 mM) were also added to the appropriate test wells. Following a 48 hr incubation cells were trypsinized, pelleted and resuspended in 200 µl of culture media to which an equal volume of trypan blue was added. Cells excluding typan blue were scored in triplicate using a haemocytometer and a light microscope. Prior to experimentation oxamate and DCA were both tested for toxicity at a range of treatment concentrations. For each inhibitor, a treatment concentration was selected that conferred little to no toxicity in cells (20 mM oxamate, 2.5 mM DCA).

### Fluorescence microscopy

Mitochondrial ROS production was visualized by the fluorescent dye Mitotracker Red CM-H2XRos (MTR). Stock MTR was dissolved in dimethylsulfoxide (DMSO) at a concentration of 1 mM and stored at −20°C. PC12 and B12 cells were plated (8×10^4^) in 6 well glass bottom tissue culture dishes pretreated with polylysine (50 µg/ml for 3 hr) and incubated overnight. PC12 parental cells were plated at a higher density of 3×10^5^ cells/well. The following day cells were treated with 20 µM Aβ_25–35_ for 48 hr. Following treatment with Aβ, the media was aspirated and new media was added containing MTR at a concentration of 100 nM. Plates were then incubated at 37°C for 20 min, washed in DPBS containing Hoecsht stain (10 µg/ml), followed by an additional wash in DPBS and then placed in phenol red free DMEM. Cells were visualized by fluorescence microscopy (Zeiss-AxioObserver, 40X objective) and pictures were taken using a Q Imaging (Retiga 1300 monochrome 10-bit) camera and Q Capture software. Pictures were taken of three random fields of view for each experiment. MTR fluorescence was quantified with ImageJ software.

### Derivation of PDK1 and LDHA knockdown cell lines

One resistant clone from each cell line, R7 (PC12) and R2 (B12) were selected to make stable knock down lines. All cells were transfected with lipofectamine (Invitrogen, Carlsbad, CA, USA) according to the manufacturer's directions. Cells were plated at a density to achieve 70–80% confluency. Cells were transfected with HuSH 29mer shRNA (Origene, Rockville, MD, USA) constructs directed at rat *Ldha* or rat *Pdk1*. The shRNA vector is cloned in the pRS plasmid under the control of the U6 promoter for mammalian cell expression. For each specific mRNA, 2 shRNA's containing expression cassettes, targeted at different parts of the mRNA were selected. The selected shRNA constructs directed against *Ldha* were: 71-TGGAATCTCAGATGTTGTGAAGGTGACAC and 72–CTTGTGCCATCAGTATCT-TAATGAAGGAC); the shRNA constructs directed against *Pdk1* were: 29-AATCACCAGGACAGCCAATACAAGTGGTT and 30-TCGGTTCTACATGAGTCGCATC-TCAATTA). A non effective shRNA construct (scrambled) in pRS plasmid was selected as a negative control. Each shRNA construct for both *Ldha* and *Pdk1* in addition to the scrambled construct (5 total) were separately transfected and selected for their resistance to 1 µg/ml puromycin. Approximately 10–12 clones were picked, expanded and screened by western blot analysis for successful knockdown.

### Statistical Analysis

Data are presented as means ± SD resulting from a least three independent experiments. Data were analyzed statistically using a one-way ANOVA followed by a Tukey test (VassarStats). Results were considered statistically significant at P<0.05.

## Results

### Increased LDHA activity and lactate production in Aβ-resistant cells

**Figure 1:**
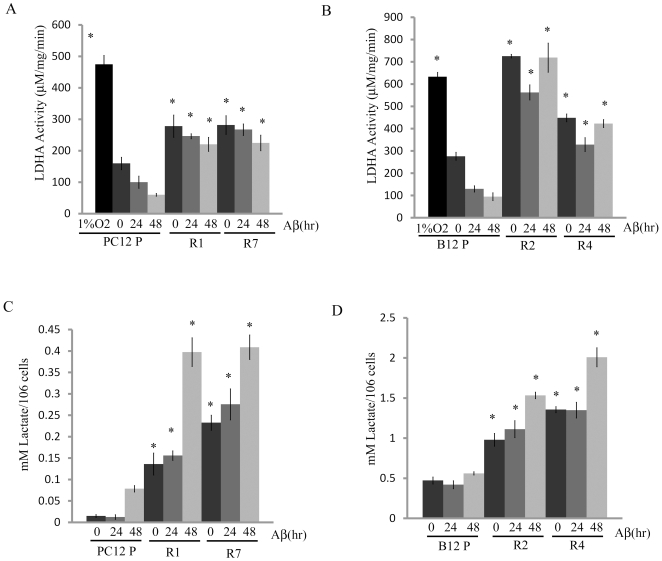
LDHA activity and lactate levels are elevated in Aβ-resistant cells. **A**) LDHA activity was significantly greater in PC12 Aβ resistant cells lines, R1 and R7, as compared to the parental line both in the absence and presence of Aβ (*, P<0.01). As a control, PC12 parental cells exposed to 24 hr hypoxia (1%O_2_) also exhibited significantly greater LDHA activity when compared to untreated parental cells (*, P<0.01). **B**) LDHA activity was also significantly greater in B12 Aβ resistant cells lines, R2 and R4, as compared to the parental line under the same conditions (* P<0.01). B12 parental cells exposed to 24 hr hypoxia (1%O_2_) also exhibited greater LDHA activity when compared to untreated parental cells (*, P<0.01). (**C**) Extracellular lactate was significantly elevated in PC12 Aβ-resistant lines, R1 and R7 and (**D**) B12 Aβ resistant lines, R2 and R4, when compared to their respective parental cells cultured under similar conditions (*; P<0.01). Data represent the average ± SD of three independent experiments. Data was analyzed by a one-way ANOVA followed by a Tukey test.

LDHA is responsible for the conversion of pyruvate to lactate and the increased activity of this enzyme in cancer cells is strongly associated with the Warburg effect [41], [42], [43]. To determine if a similar effect is observed in Aβ-resistant cells, we measured LDHA activity and secreted lactate levels in both Aβ-sensitive PC12 and B12 parental cells and clonal lines that have been selected for resistance to Aβ. Interestingly, all the resistant lines displayed significantly higher levels of LDHA activity as compared to their parental counterparts, with or without exposure to Aβ (Fig. 1 A, B, P<0.01). Additionally, the activity of LDHA in both parental lines decreased with increased exposure to Aβ. However, the activity of LDHA in the PC12 resistant lines, R1 and R7, appeared to decrease only slightly with exposure to Aβ, but remained significantly greater than the parental line for all treatments (P<0.01). Similarly, the activity of LDHA in the B12 resistant lines, R2 and R4, remained significantly greater than the parental line with or without exposure to Aβ (P<0.01). As a control, PC12 and B12 parental cells were exposed to hypoxic conditions (1% O_2_) for 24 hr, as hypoxia is a known activator of LDHA [16], revealing that both lines exhibited increased LDHA activity when exposed to hypoxic conditions. We also examined LDHA protein levels by western blot analysis using an LDHA-specific antibody. However, there was no clear trend in LDHA expression between both PC12 and B12 parental and resistant lines (data not shown). LDHA activity has been shown to be regulated by both allosteric effects and post translational modifications [44], [45], [46], [47], therefore the increased activity we observed in Aβ resistant cells may be attributed to these modifications.

LDHA catalyzes the conversion of pyruvate to lactate which is then released to the extracellular space by the monocarboxylate transporters (MCT). To determine if the elevated LDHA in Aβ resistant cells was associated with increased lactate production, the concentration of lactate in the extracellular media of cultured B12 and PC12 cells was examined. A significant increase in the concentration of lactate was detected in the extracellular media of PC12 and B12 resistant lines when compared to their respective parental lines at 24 hr and 48 hr, with or without exposure to Aβ (20 µM) (Fig. 1 C, D, P<0.01). The concentration of lactate in both PC12 and B12 parental lines decreased with exposure to Aβ whereas the concentration of lactate was maintained or increased in all the resistant lines when exposed to Aβ for 24 or 48 hrs. These findings indicated that increased LDHA activity and associated lactate production is strongly associated with Aβ-resistance.

### PDK1 levels are elevated in Aβ-resistant cells

**Figure 2:**
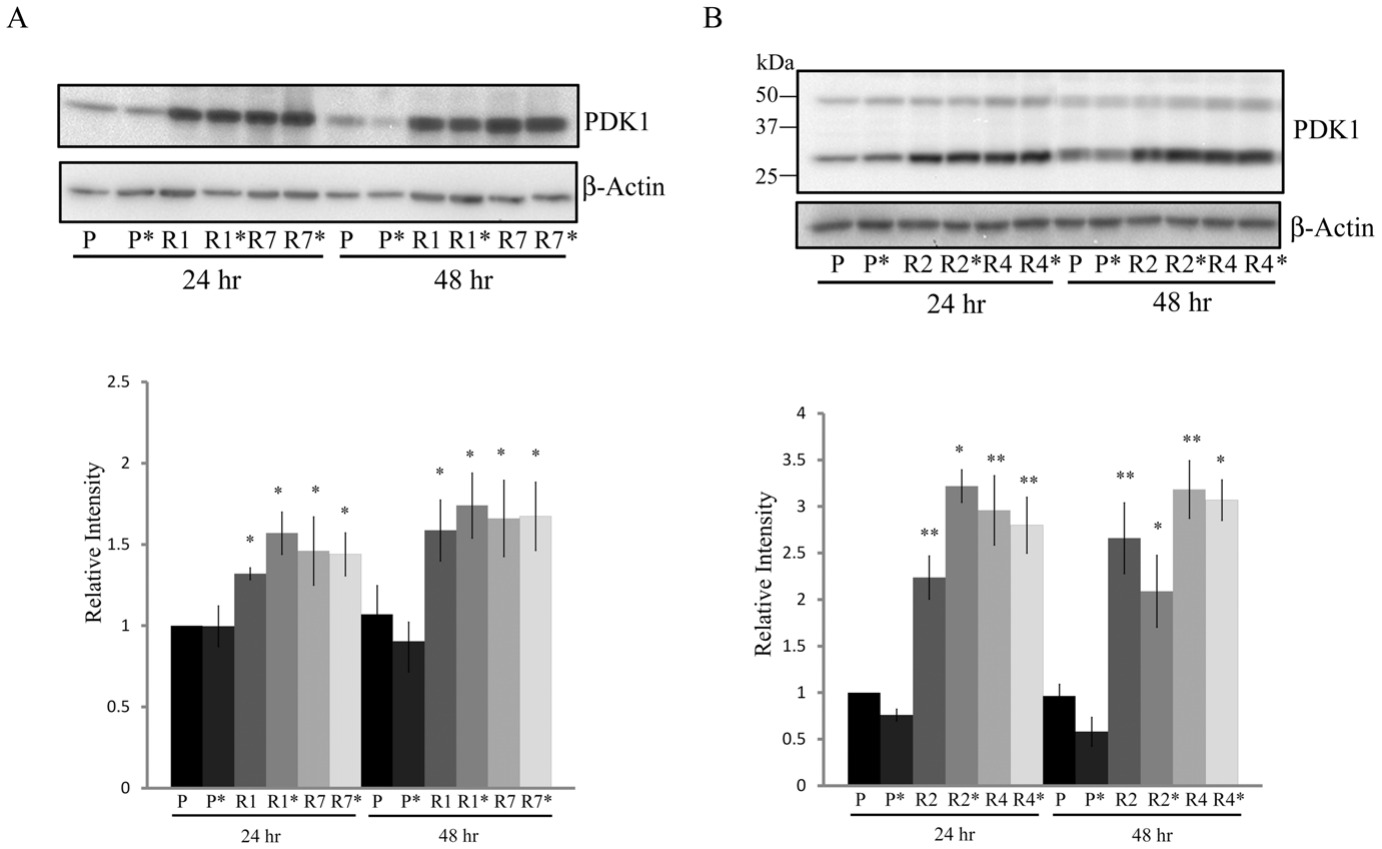
PDK1 is upregulated in Aβ resistant cells. **A**) PDK1 levels were significantly elevated in both PC12 resistant lines, R1 and R7, when compared to the parental cell line (*,P<0.01). These elevated levels were maintained following 24 and 48 hr Aβ treatment (* 20 µM). **B**) PDK1 levels were also significantly greater in both B12 resistant lines, R2 and R4, when compared to the parental line (*,P<0.01; **,P<0.05). These increased levels were also maintained with Aβ treatment. An additional band of approximately 30 kDa that was more prominent than full length PDK1 (∼48 kDa). This additional band also showed the same elevated trend as full length PDK1 in the R2 and R4 lines when compared to the parental under similar conditions. The smaller band may represent a cleavage product of PDK1. Densitometric analysis of PDK1 band densities relative to actin are found in the lower panel. Relative intensity was calculated by comparing the PDK1/actin ratio of the resistant lines to the same ratio in the parental cell line. Data represent the average ± SD of three independent experiments.

Elevated PDK1 expression in cancer cells has been shown to result in reduced mitochondrial respiration and resistance to apoptosis [22]. To determine if elevated PDK1 expression contributes to resistance to Aβ toxicity, cell extracts from sensitive and resistance cells were analyzed by Western blot analysis. Highly elevated PDK1 levels were detected in all Aβ-resistant lines relative to parental cell lines in either the presence of absence of Aβ (Fig. 2). Interestingly, an additional band around 30 kDa was detected that was more prominent than full length PDK1 (∼48 kDa) in B12 cells. This lower molecular weight band also showed the same elevated trend as full length PDK1 in the R2 and R4 lines (Fig. 2 B). The lower molecular weight PDK1 band may represent some form of cleavage product/posttranslational modification of PDK1. In contrast, the PC12 and B12 parental cell lines exhibited a 14% and 39.5% reduction in PDK1 levels respectively following 48 hr exposure to Aβ. Thus, increased PDK1 expression strongly correlates with Aβ resistance.

### Mitochondrial ROS production is decreased in Aβ-resistant cells

**Figure 3:**
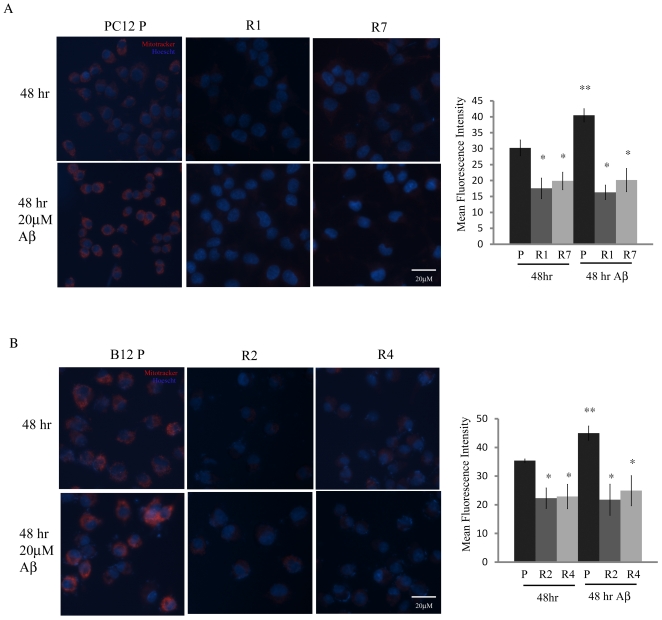
Decreased mitochondrial reactive oxygen species in Aβ resistant cells. **A**) PC12 Aβ resistant lines R1 and R7 exhibited a significant reduction in mitochondrial reactive oxygen species (ROS) compared to the parental cell line under normal culture conditions (*, P<0.01). This decrease in ROS was maintained with 48 hr Aβ (20 µM) exposure. Conversely, mitochondrial ROS significantly increased in the parental line when exposed to Aβ (**, P<0.05). **B**) The B12 resistant lines, R2 and R4 also exhibited decreased mitochondrial ROS when compared to parental cells under similar conditions (*, P<0.01). A similar increase in mitochondrial ROS in B12 parental cells was also observed following treatment with Aβ for 48 hr (**, P<0.05). Cells were stained with MitoTracker Red (100 nM) and nuclei were stained with Hoescht (10 µg/ml) and visualized by fluorescence microscopy at 400X magnification and quantified with ImageJ software. Data represent the average ± SD of three independent experiments. Data was analyzed by a one-way ANOVA followed by a Tukey test.

A decrease in mitochondrial-derived ROS is a key feature of the Warburg effect and plays a prominent role in resistance to apoptosis [22]. We therefore examined mitochondrial ROS in Aβ sensitive and resistant cells using Mitotracker ROS Red (MTR); a dye that specifically incorporates in mitochondria and only fluoresces in the presence of ROS [22]. A significant decrease in mitochondrial ROS, as measured by mean MTR fluorescence, was observed in PC12 and B12 resistant cells compared to their parental counterparts with or without exposure to Aβ (20 µM) (Fig. 3 A, B, P<0.01). In contrast, there was a pronounced increase in mitochondrial ROS in both PC12 and B12 parental lines following 48 hr exposure to Aβ (P<0.05). Thus Aβ-resistant cells appear to have an altered metabolism resulting in significantly less mitochondrial-derived ROS under both basal and stressed conditions.

### Chemical inhibition of LDHA or PDK1 restores sensitivity to Aβ in resistant cells

**Figure 4:**
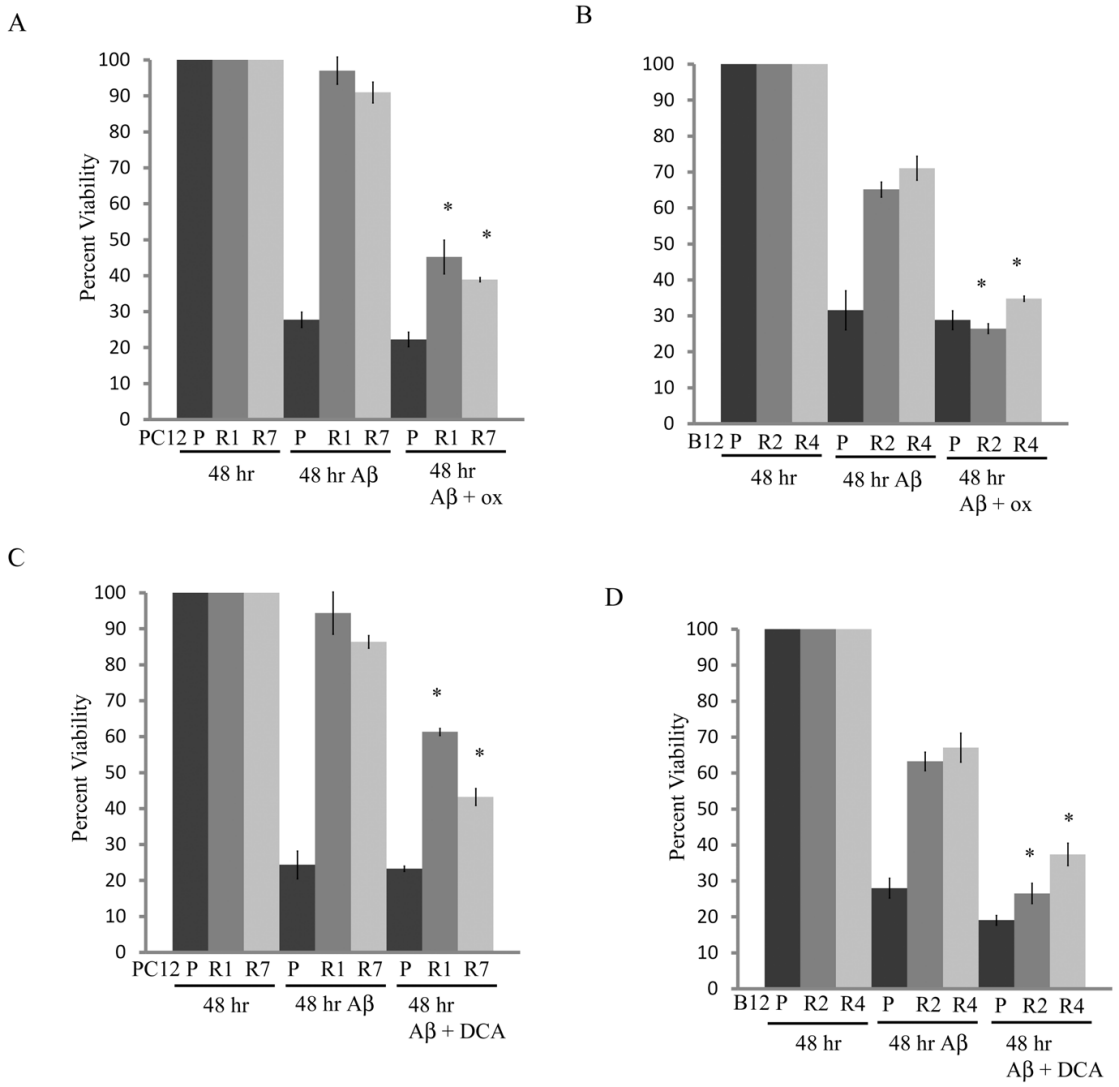
Chemically inhibiting LDHA or PDK1 decreases cell viability in Aβ resistant cells. A significant decrease in cell viability in both PC12 (**A**) and B12 (**B**) resistant lines was observed after 48 hr concomitant exposure to Aβ (20 µM) and 20 mM oxamate (ox), a chemical inhibitor of LDHA (*P<0.01). Similarly, 48 hr Aβ exposure significantly decreased cell viability in both PC12 (**C**) and B12 (**D**) resistant lines when cells were co-treated with 2.5 mM dichloroacetate (DCA), a chemical inhibitor of PDK1 (*P<0.01). Interestingly, the cell viability of the parental lines does not appear to decrease with treatment of either inhibitor and Aβ. Cell viability was determined by the reduction of the tetrazolium salt MTT. Data are representative of three separate experiments. Data was analyzed by a one-way ANOVA followed by a Tukey test.

Inhibition of LDHA or PDK1 using the chemical inhibitors oxamate and dichloroacetate (DCA) respectively, has been shown to counter the Warburg effect and apoptosis resistance in cancer cells [22], [27], [48]. When the PC12 and B12 resistant cell lines were treated with either chemical inhibitor, oxamate (20 mM) or DCA (2.5 mM), and Aβ (20 µM) there was a significant decrease in cell viability when compared to Aβ treatment alone (Fig. 4 A–D, P<0.01). Interestingly, exposure of the PC12 and B12 parental lines to either oxamate or DCA did not appear to potentiate Aβ toxicity. To ensure that oxamate and DCA did not interfere with the reduction of MTT and subsequent formazan formation, these experiments were repeated using a trypan-blue exclusion viability assay and similar results were observed (Figure S1). These findings indicate that chemical inhibition of either LDHA or PDK1 can resensitize Aβ-resistant cells to Aβ.

### Attenuated LDHA and PDK1 expression reverses Aβ resistance

**Figure 5:**
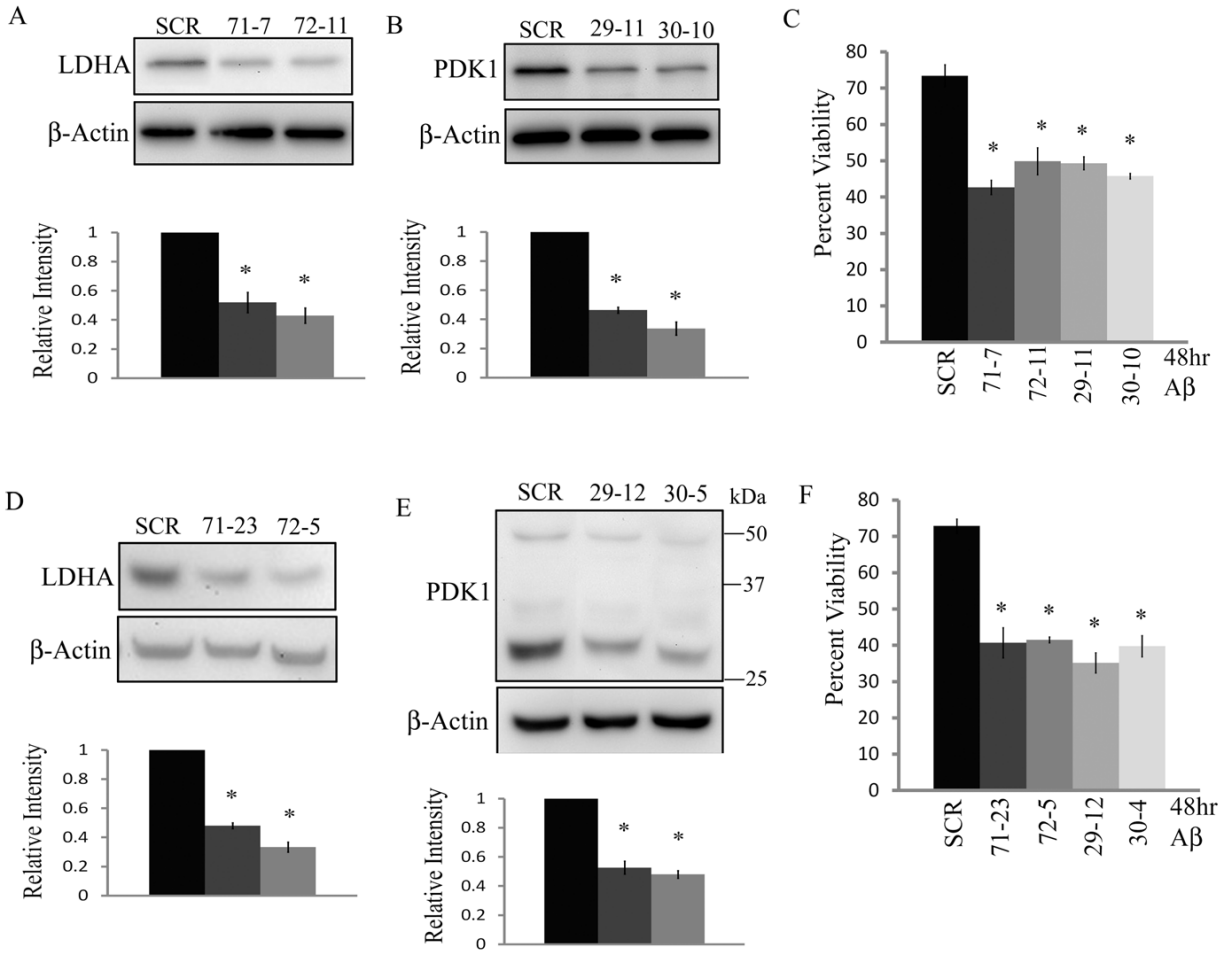
Attenuated LDHA or PDK1 expression increases sensitivity of resistant cell lines to Aβ. **A**) Immunoblot analysis of PC12 R7 resistant cells stably transfected with LDHA-specific shRNA vectors revealed two clonal cell lines (clones 71-7,72-11) exhibited a significant decrease in LDHA protein levels when compared to an R7 cell line transfected with a non-specific shRNA (SCR) (*P<0.01). **B**) Immunoblot analysis also confirmed a significant decrease in PDK1 protein in two PDK-1 shRNA stably transfected R7 cell lines, (clones 29-1 and 30-10) when compared to the control cell line (*P<0.01). **C**) A significant decrease in the cell viability of the R7 clones with attenuated expression of either LDHA or PDK1 when exposed to Aβ (20 µM) for 48 hr when compared to the control (* P<0.01). **D**) Immunoblots of R2 cell lines (clones 71-23 and 72-5) confirming significantly decreased LDHA expression when compared to a control cell line (SCR) (*P<0.01). **E**) R2 clonal cell lines, (clones 29-12 and 30-4) stably expressing PDK-1 shRNA showed a significant decrease in the PDK1 full length protein (∼48 kDa) as well as a decrease in the proposed PDK1 cleavage product (∼30 kDa) when compared to the control (*P<0.01). **F**) Both R2 LDHA and PDK1 knockdown cell lines exposed to 48 hr Aβ (20 µM) treatment showed a significant decrease in cell viability when compared to R2 control (* P<0.01). Densitometric analysis of LDHA and PDK1 band densities relative to actin are found below the corresponding blot. Relative intensity was calculated by comparing the LDHA/actin or PDK1/actin ratio of the resistant lines to the same ratio in the scrambled shRNA cell line. Data represent the average ± SD of three independent experiments. Data was analyzed by a one-way ANOVA followed by a Tukey test.

Because chemical inhibitors such as oxamate and DCA could potentially have off-target effects, we sought to determine if specific inhibition of LDHA and PDK1 expression by shRNA –mediated knockdown could also render resistant cells sensitive to Aβ. Immunoblot analysis confirmed that PC12 R7 and B12 R2 cell lines stably transfected with shRNA vectors containing sequences directed at rat *Ldha* or *Pdk1* transcripts exhibited decreased expression of the targeted mRNAs compared to cells transfected with a control shRNA containing a non-specific/scrambled (scram) sequence (Fig. 5). Densitometric analysis revealed there was a 52% and 62% reduction in LDHA levels in the R7 knockdown lines 71-7 and 72-11 respectively, and a 65% and 72% reduction in PDK1 levels in the R7 knockdown lines 29-11 and 30-10 respectively when compared to the R7 control line scram-10 (Fig. 5 A, B). By knocking down either LDHA or PDK1 expression in the R7 Aβ resistant cell line, we observed a significant decrease in cell viability, following exposure to 48 hr Aβ (20 µM) when compared to the control (Fig. 5 C, P<0.01). Reduction of LDHA or PDK1 expression in R7 cells resulted in a 40 to 50% reduction in viability compared to the control cell lines. (Fig. 5 C).

Western blot analysis revealed there was a 50% and 62% reduction in LDHA levels in the R2 knockdown lines 71-23 and 72-5 respectively, and a 48% and 49% reduction in PDK1 levels in the R2 knockdown lines 29-12 and 30-4 respectively, when compared to R2 line scram 5 (Fig. 5 D, E). Exposure of the R2 LDHA or PDK1 knockdown clones to 48 hr Aβ (20 µM) resulted in a significant reduction in cell viability (between 43 to 52%) when compared to the control (Fig.5 F, P<0.01). Thus, specifically targeting either LDHA or PDK1 expression in Aβ resistant cells results in re-sensitization to Aβ and increased death.

## Discussion

Understanding how nerve cells become resistant to Aβ toxicity is central to understanding how some nerve cells within the AD brain are able to survive while large numbers of cells die. It had been previously shown that Aβ resistant cells, through increased HIF-1 activity, modulate their cell metabolism to increase glucose uptake and glycolysis in the presence of Aβ [14]. Additionally, HIF-1 activation in Aβ sensitive cells is sufficient for neuroprotection against Aβ [14]. However, this earlier study did not precisely define how HIF-1 functions to protect cells from Aβ toxicity. One compelling idea is that HIF-1 driven alteration in metabolism in Aβ resistant cells confers a selective advantage for survival in the hostile environment of the AD brain [14], [49]. Increased HIF-1 activity has been shown to enhance transcription of both *LDHA* and *PDK1* [16], [18]. In this study we observed that Aβ resistant cells have increased LDHA activity and PDK1 expression, both in the absence or presence of Aβ, and these events correlate with decreased mitochondrial derived ROS.

The question arises as to why decreased mitochondrial respiration in Aβ resistant cells would be advantageous? A previous study showed that cells depleted of mitochondrial DNA, lacking critical catalytic subunits of the respiratory chain and incapable of mitochondrial respiration, were unaffected by Aβ exposure [50]. Mitochondria are the major site of ROS production, which is believed to be a major factor leading to Aβ induced death [7]. This is supported by the observation that exogenously applied antioxidants protect primary CNS cultures and clonal lines from Aβ toxicity [7]. In addition, overexpression of the mitochondrial antioxidant enzyme manganese superoxide dismutase (MnSOD) in AD transgenic mice increases resistance to Aβ and attenuates the AD phenotype [51]. These studies suggest that functional mitochondria are required for Aβ to elicit a toxic effect.

**Figure 6:**
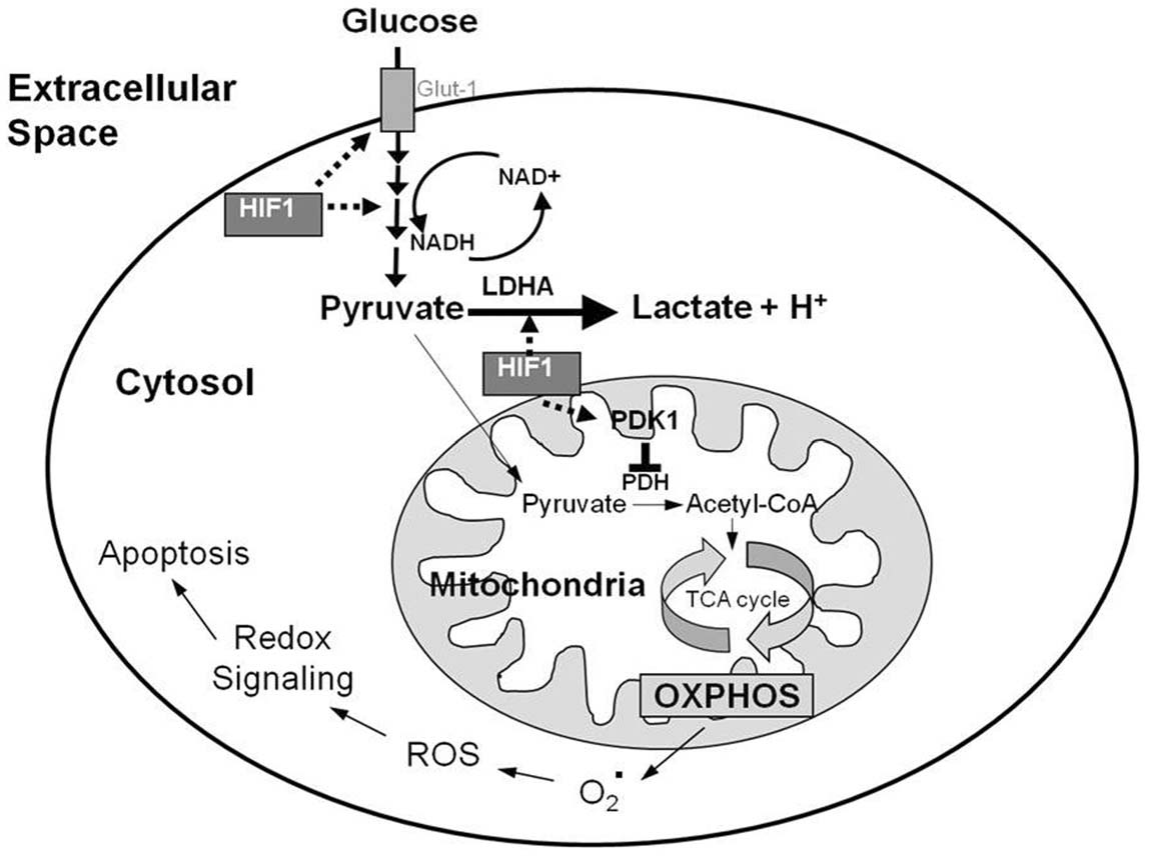
Proposed role of the Warburg effect in Aβ resistant cells. The stabilization of hypoxia inducible factor 1 α (HIF1α) and subsequent increased activity of HIF-1 in amyloid beta (Aβ) resistant cells stimulates increased expression of glucose transporters (Glut1), and glycolytic enzymes increasing the conversion of glucose to pyruvate. Additionally, HIF-1 promotes the reduction of pyruvate to lactate through the upregulation of lactate dehydrogenase A (LDHA). HIF-1 also actively suppresses the production of acetyl CoA through the mitochondria via increased expression of pyruvate dehydrogenase kinase 1 (PDK1), which phosphorylates and inhibits pyruvate dehydrogenase (PDH) resulting in decreased flux through the tricarboxcylic acid (TCA) cycle and repressed oxidative phosphorylation (OXPHOS). The decrease in electron transport activity decreases the generation of reactive oxygen species (ROS) in the mitochondria and renders cells more resistant to apoptosis in the presence of Aβ.

In support of these findings, the data in this study reveal that Aβ resistant nerve cell lines exhibit metabolic reprogramming and decreased mitochondrial ROS which contribute to their resistance against Aβ toxicity. Interestingly, these low levels of ROS were maintained even after exposure to Aβ. In contrast, the parental sensitive lines showed elevated mitochondrial ROS with exposure to Aβ. The increase in LDHA activity, lactate production and PDK1 levels, and decrease in mitochondrial ROS strongly indicate that a Warburg effect exists in Aβ resistant cells. Interestingly chemical or genetic manipulation of either LDHA or PDK1 resulted in increased sensitivity to Aβ in all resistant cell lines. These observations indicate that both enzymes, LDHA and PDK1, contribute to resistance to Aβ. However, inhibiting or knocking down either enzyme resulted in similar levels of sensitivity to Aβ suggesting that both enzymes appear to play an equal and possibly redundant role in the resistance pathway. Knocking down or chemically inhibiting LDHA or PDK in various cancers shifts metabolism from glycolysis and lactate production to mitochondrial respiration, with an associated increase in mitochondrial ROS production and induction of apoptosis [22], [27], [42], [43]. Furthermore, inhibition of PDK1 in cancer cells *in vitro* and *in vivo* results in a reduction of phosphorylated PDH and HIF-1α levels [52]. Moreover, inhibition of PDK1 expression has been shown to decrease lactate levels and HIF-1α expression and reduce the malignant phenotype of cancer cells [52]. Therefore an increase in PDK1 and lactate production may aid in sustaining the Warburg effect through a positive feedback mechanism. We propose that HIF-1 activation of the glycolytic genes, *ldha* and *pdk1* functions to suppress apoptosis in Aβ resistant cells by decreasing mitochondrial respiration and ROS production (Fig. 6).

In the past, aerobic glycolysis within the brain has been given little attention, despite early observations suggesting that a basal level of aerobic glycolysis occurs in specific areas of the brain [53], [54]. In a healthy adult, levels of aerobic glycolysis can range from ∼10–15% of the glucose utilized by the human brain [53], [54], [55]. More recently, PET imaging revealed a significant increase in aerobic glycolysis in specific areas of the brain [56]. Specifically, the levels of aerobic glycolysis are elevated in the medial and lateral parietal and prefrontal cortices [56]. Recent PET imaging studies, using a radiotracer with a high affinity to Aβ plaques, revealed a strong correlation between the spatial distribution of aerobic glycolysis and Aβ plaques in both patients with AD, as well as cognitively normal patients with high levels of Aβ deposition but without clinical manifestation of the disease [28]. Additionally, the spatial distribution of Aβ deposition and increased aerobic glycolysis closely mirrors the distribution of aerobic glycolysis in the normal healthy brain [28], [56]. Thus one could conclude that areas of increased aerobic glycolysis within the brain are more susceptible to Aβ accumulation. However, the opposing viewpoint is that an increase in aerobic glycolysis is an innate protective response that limits the toxicity of Aβ. Areas within the normal brain that show increased aerobic glycolysis highly overlap with the ‘default mode network'- brain regions that are most active when an individual is awake but not engaged in a specific task [28], [56]. We propose that these areas of the brain are the most susceptible to insult in AD and thus exhibit a Warburg effect as a protective mechanism or a built in resistance mechanism that can be further activated in the presence of high levels of Aβ.

Although a Warburg effect aids in the survival against Aβ toxicity, it may have a few detrimental effects. First, a reliance on glycolysis, an inefficient means of producing energy, increases the demand for glucose in these cells. Thus a small decrease in glucose availability may render these cells incapable of producing sufficient energy to sustain their function and lead to cell death. Secondly, an increase in lactate production may result in decreased glutathione levels, the major antioxidant in the brain responsible for detoxifying ROS [57]. Lactate has been shown to inhibit the enzymatic steps of glutathione synthesis, thus an increase in lactate would likely result in a decrease in glutathione synthesis and levels, which could result in a decreased tolerance to ROS [57]. Lastly, HIF-1 mediated increase in glycolysis may result in an increase in Aβ production and deposition within the brain. HIF-1 has been shown to transcriptionally regulate both the β-site APP cleaving enzyme (β-secretase/BACE1) and an essential enzyme involved in the presenilin/γ-secretase complex anterior pharynx-defective 1A (APH-1A)[58], [59]. Hypoxia or overexpression of HIF-1α increases the BACE1 mRNA and protein levels in mouse neuroblastoma N2a cells [58]. Similarly, treatment of HeLa cells stably expressing the human APP Swedish mutation with NiCl_2_ (a chemical mimic of hypoxia) results in an increase in APH-1A mRNA and protein expression accompanied by an increased secretion of Aβ [59]. These findings have quelled enthusiasm for the treatment of AD using metal chelators to enhance HIF1α activity. However, our findings suggest that enhancement of PDK1 activity alone may offer a better neuroprotective strategy as it is unlikely to affect APP processing. Further studies will need to be conducted to examine this hypothesis.

### Conclusion

We have shown for the first time a Warburg effect exists in Aβ resistant nerve cell lines, and contributes to resistance against Aβ toxicity. These findings suggest that the Warburg effect may act as a common resistance mechanism in a variety of cell types in response to diverse environmental stresses. However the physiological relevance of these findings in an *in vivo* AD model remains unknown. This shift in cell metabolism to rely heavily on glycolysis and lactate production for energy needs may provide nerve cells with a mechanism to overcome the pro-oxidant conditions elicited by Aβ exposure. Chemical means of modulating this pathway may be of therapeutic interest in the treatment of AD. Moreover, characterization of the mechanisms by which glycolysis is upregulated in Aβ resistant cells could reveal possible targets for drug therapy in the treatment of AD.

## Supporting Information

Figure S1 **Treatment with oxamate or DCA decreases cell viability in Aβ resistant cells.** A significant decrease in cell viability in both PC12 (**A**) and B12 (**B**) resistant lines was observed after 48 hr concomitant exposure to Aβ (20 µM) and 20 mM oxamate (ox), a chemical inhibitor of LDHA, or Aβ (20 µM) and 2.5 mM dichloroacetate (DCA), a chemical inhibitor of PDK1 (*P<0.01; **P<0.05). Cell viability was determined by trypan blue exclusion. Data are representative of three separate experiments. Data was analyzed by a one-way ANOVA followed by a Tukey test.
(TIF)
