# Supplementary figures and images for: Amyloid Beta Resistance in Nerve Cell Lines Is Mediated by the Warburg Effect
Source: PLoS One. 2011 Apr 26;6(4):e19191. doi: 10.1371/journal.pone.0019191 (PMC3082554; doi:10.1371/journal.pone.0019191)

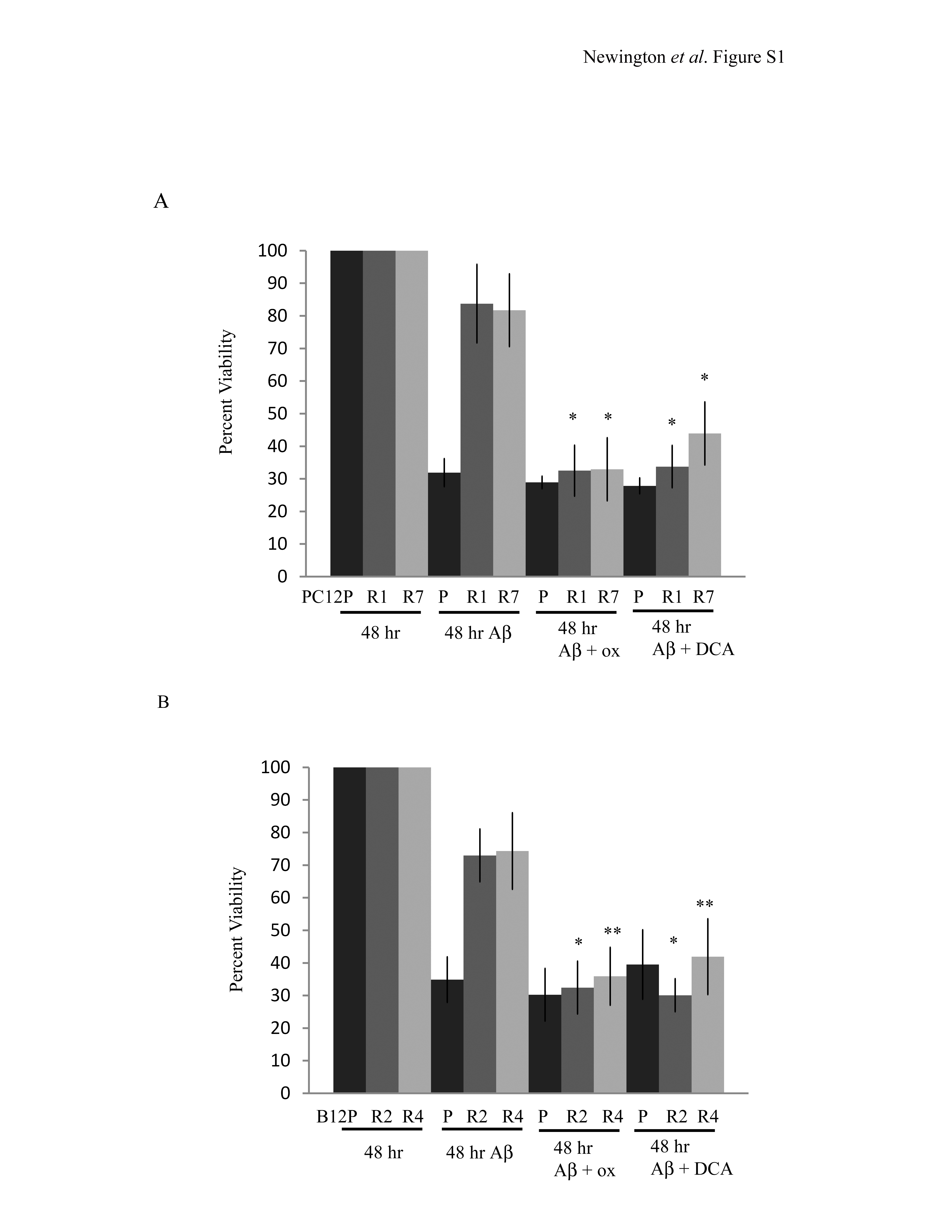

Supplement: Figure S1 — Treatment with oxamate or DCA decreases cell viability in Aβ resistant cells. A significant decrease in cell viability in both PC12 (A) and B12 (B) resistant lines was observed after 48 hr concomitant exposure to Aβ (20 µM) and 20 mM oxamate (ox), a chemical inhibitor of LDHA, or Aβ (20 µM) and 2.5 mM dichloroacetate (DCA), a chemical inhibitor of PDK1 (*P<0.01; **P<0.05). Cell viability was determined by trypan blue exclusion. Data are representative of three separate experiments. Data was analyzed by a one-way ANOVA followed by a Tukey test. (TIF) [file pone.0019191.s001.tif]
